# Impaired dendritic cell functions in lung cancer: a review of recent advances and future perspectives

**DOI:** 10.1186/s40880-019-0387-3

**Published:** 2019-07-15

**Authors:** Jing-Bo Wang, Xue Huang, Fu-Rong Li

**Affiliations:** 1 Translational Medicine Collaborative Innovation Center, The Second Clinical Medical College, Shenzhen People’s Hospital, Jinan University, 1017 Dongmen Road North, Shenzhen, 518020 Guangdong P. R. China; 2 Shenzhen Cell Therapy Public Service Platform, Shenzhen, 218020 Guangdong P. R. China

**Keywords:** Lung cancer, Dendritic cell, Immune regulation, Immunotherapy

## Abstract

Lung cancer is the leading cause of cancer mortality worldwide. Dendritic cells (DCs) are the key factors providing protective immunity against lung tumors and clinical trials have proven that DC function is reduced in lung cancer patients. It is evident that the immunoregulatory network may play a key role in the failure of the immune response to terminate tumors. Lung tumors likely employ numerous strategies to suppress DC-based anti-tumor immunity. Here, we summarize the recent advances in our understanding on lung tumor-induced immunosuppression in DCs, which affects the initiation and development of T-cell responses. We also describe which existing measures to restore DC function may be useful for clinical treatment of lung tumors. Furthering our knowledge of how lung cancer cells alter DC function to generate a tumor-supportive environment will be essential in order to guide the design of new immunotherapy strategies for clinical use.

## Introduction

Lung cancer is the leading cause of cancer death around the world [1]. More than 85% of lung cancers are non-small-cell lung cancer (NSCLC) [2]. The 5-year overall survival rate for patients with lung cancer is less than 15% and that for patients with NSCLC clinically diagnosed as stage IV is less than 5% [3]. The most common treatment for lung cancer, such as chemotherapy and radiotherapy, has shown limited effectiveness in preventing tumor progression. It is believed that recurrence after surgical resection and chemotherapy is the main cause of lung cancer death [4, 5]. Therefore, improving both diagnostic and therapeutic methods is essential for improving public health with respect to such relapses.

Developing immunotherapy strategies that can induce long-term protective immune responses against tumor-associated antigens is an emerging research topic. Such therapeutic strategies are especially vital when conventional therapies become ineffective [6]. Recent advances in immunotherapy for lung cancer include targeting costimulatory blockade and immune cell-based vaccination [7–9]. A blockade of the immune checkpoint markers, such as programmed cell death 1 (PD-1), programmed cell death 1 ligand 1 (PD-L1) and cytotoxic T-lymphocyte-associated antigen-4 (CTLA4), resulted in a significantly prolonged survival rate, indicating a systemic anti-tumor immune deficiency in lung cancers [10–12]. However, the expression of these immune checkpoint markers differs from one cancer to another, limiting the general application of the approaches targeting them. For example, patients with low PD-1 expression have poor responses to anti-PD-1 treatment [12–14]. For this reason, other immunotherapeutic strategies must be developed to promote consistent therapeutic effects.

Dendritic cells (DCs) are crucial for the activation of antigen-specific CD8 T lymphocytes, a pivotal step in the initiation of the innate and adaptive immune responses, which are essential for tumor cell clearance. Previous studies have demonstrated that PD-1-deficient DCs had a stronger ability to induce antigen-specific CD8^+^ T cell proliferation than wild-type DCs in vivo [15]. As a nano-sized vesicle, exosomes derived from different cell types selectively enrich the proteins associated with specific cell functions [16, 17]. Moreover, DC-derived exosomes can be used for maintenance immunotherapy in NSCLC patients whose disease responded or were stabilized after induction chemotherapy, as previously described [18]. Thus, DC mobilization may be an effective treatment strategy for cancer [19, 20]. Anti-tumor effects of DCs can be reduced by several factors, including low DC count, low antigen presentation efficiency of tumor-infiltrating DCs, and weak ability of DC to migrate into tumor mass [21, 22]. A previous study has shown that the maturation rate of DCs in patients with lung tumors was significantly lower than that in healthy controls [23]. In addition to enhancing the antigen-presenting ability of DCs, blockade of the immunosuppression signal between lung tumor cells and DCs is also essential for the development of DC-based anti-tumor therapies. In this review, we summarized the mechanisms involved in lung cancer-induced DC inhibition and the recent advances in DC-based immunotherapy. Additionally, we addressed the potential approaches for restoring DC function in lung cancers, which is the key for designing more successful DC-based anti-tumor therapy.

## Origin of DCs

Myeloid cells include different types of innate immune cells that can clear damaged cells and promote the recruitment of immune effector cells. In the tumor microenvironment (TME), tumor-infiltrating myeloid cells (TIMs) play a major role in anti-tumor response [24, 25]. TIMs mainly consist of granulocytes and mononuclear phagocytes. These cells share the ability to present tumor-associated antigens to T cells, which are closely related to tumor progression and response to immunotherapy [26]. Among all TIMs, DCs are best equipped to activate T cells.

DCs are professional antigen-presenting immune cells and are distributed throughout the body. They originate from the bone marrow, circulate in the blood, and have two ultimate fates, either enter the lymphoid nodes to act as lymphoid DCs or enter peripheral tissues to differentiate into non-lymphoid DCs [27]. DCs are generated from both lymphoid and myeloid progenitors in the bone marrow, which produce conventional DCs (cDCs) and plasmacytoid DCs (pDCs), respectively, in adoptive transfer experiments [28]. Among hematopoietic stem cells, monocyte-DC progenitors (MDPs) can give rise to common myeloid progenitors (CMPs), including a subset of CMPs that express colony stimulating factor 1 receptor (FMS)-like tyrosine kinase 3 (FLT3) [28]. FLT3 expression in CMPs is required to maintain cDC developmental potential [29]. MDPs can also give rise to common monocyte progenitors (cMoPs) that differentiate into monocytes. Ly6C+ circulating monocytes can differentiate into monocyte-derived DCs (MoDCs) under the appropriate circumstances [30, 31]. In clinical trials, MoDCs were shown to be easy to be induced from patient’s peripheral blood monocytes and were capable of inducing tumor-specific immune responses when co-cultured with the corresponding antigens [32].

**Fig. 1:**
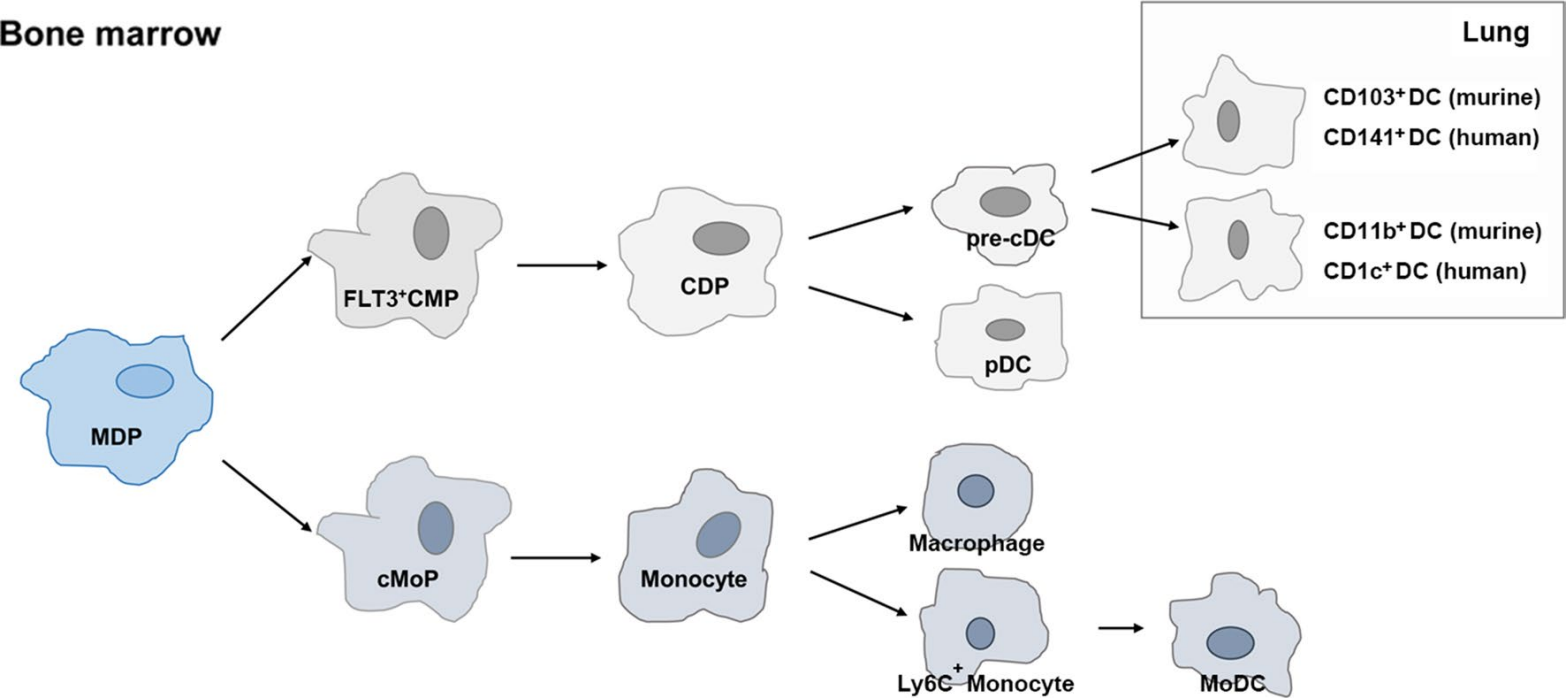
Origin of DCs. MDPs among hematopoietic stem cells give rise to common FLT3-expressing CMPs, which are the precursors of pre-cDCs and pDCs. Monocytes can differentiate into MODCs under the appropriate circumstances and Pre-cDCs circulate into lung tissue and differentiate into different classes of DCs. MDP: Monocyte-DC progenitors; FLT3: colony stimulating factor 1 receptor (FMS)-like tyrosine kinase 3; pDC: plasmacytoid DC; cDC: conventional DC; MODCs: monocyte-derived DCs: CMPs: common myeloid progenitors

Several DC subsets which can be broadly divided into cDCs and pDCs have been identified. Detailed analyses of murine and human lungs have revealed that two main subsets of cDCs are present in a steady state [33]. In murine lungs, these two subsets express CD103 and CD11b, respectively. CD103^+^ cDCs are regulated by interferon regulatory factor 8 (IRF8) and CD11b^+^ cDCs are regulated by IRF4 [34, 35]. CD103^+^ DCs have been shown to control the activation of local CD8^+^ T cell in TME as active APCs. CD11b^+^ DCs initiate Th2- and Th17-biased immune responses, thus making only a minor contribution to tumor clearance [36, 37]. Equivalent cDC subsets in human lungs expressing CD141 and CD1c, respectively, have similar functions [38]. On the other hand, pDCs are mainly characterized by the production of large amounts of type I interferon (IFN) [39]. The expression of toll-like receptor 7 and 9 (TLR7 and TLR9) on pDCs confers antiviral activity [40]. Relative to cDCs, pDCs show less antigen-presenting ability and seem to play an important role in maintaining self-tolerance [41]. The origin of DCs is illustrated in Fig. 1.

## Lung cancer-induced DC suppression can impede immune clearance

### Functional DCs are excluded from lung tumor lesions

The antigen presenting ability of DCs plays an important role in the activation of anti-tumor T cell. DC metabolism controls T cell polarization in the lungs [42]. Under normal circumstances, original DCs infiltrate into the lung from the bone marrow and differentiate into two main subsets of mature DCs (CD11b^+^ and CD103^+^ DCs). Mature DCs express a higher level of co-stimulatory molecules (CD40/80/86), and an elevated cytokine levels indicate a potent T cell activating ability. The prominent expression of major histocompatibility complex (MHC) type II molecules on CD11b^+^ DCs facilitates the activation of CD4 T cells. In contrast, CD103^+^ DCs, which have been recently identified as active APCs in the TME, preferentially induce CD8^+^ T cell responses via MHC type I molecules [43–46]. However, compared to their counterparts in the peritumoral lung, lung tumor-infiltrating DCs were found to show increased expression of CD11b [47]. Moreover, a smaller population of CD103^+^ DCs was observed within lung tumors in a mouse model [47]. Furthermore, a paired single-cell transcriptional analysis of tumorous and non-tumorous human lungs also revealed two clusters of DCs: one cluster consisted of CD141^+^ DCs, which mainly interact with CD8^+^ T cells. The other cluster contained CD1c^+^ DCs, which have an increased potential to activate CD4^+^ T cells. Compared with non-tumorous lung tissues, the proportion of CD141^+^ DCs is significantly lower in tumorous tissues [38]. It is believed that human CD141^+^ DCs and CD1c^+^ DCs are similar to murine CD103^+^ DCs and CD11b^+^ DCs [48]. Taken together, the DC subset which could activate CD8^+^ T cell was reduced in tumor tissues. These studies demonstrate that lung cancers dynamically exclude functional DCs from the tumor region to support malignant progression.

### Immunosuppressive pDCs are recruited into the surrounding tissues of lung tumors

The key role of pDCs in innate immunity has been clearly elucidated over the past few decades [49, 50]. Previous studies have shown that inactive pDCs are present in lung cancers associated with poor patient prognosis [51, 52]. Tumor-associated pDCs show impaired type I IFN production [53]. This IFN has been shown to participate in cytotoxic, immunosuppressive, and anti-tumor responses [53, 54]. The activation of intratumoral pDCs by TLR-9 agonist may induce melanoma regression via natural killer (NK) cell-dependent pathways in a C57BL/6 melanoma model [55]. Moreover, the secretion of C–C chemokine receptor type 5 (CCR5) by pDCs contributes to NK cell recruitment in tumors [55]. cDC cross-presentation is thought to depend on NK cell recruitment [55]. However, TLR-9 activation in lung tumor-bearing mice leads to the recruitment of regulatory T cells (Tregs), thus contributing to an immunosuppressive environment around the tumor [56]. Narusawa et al. [57] used imiquimod, a TLR-7 ligand, to activate pDCs in a Lewis lung carcinoma model. The addition of imiquimod significantly improved the antitumor phenotype in pDCs and decreased the proportion of Tregs in tumor-draining lymph nodes. An earlier study using a mouse model showed that low doses of lipopolysaccharides (LPS) may increase the lung tumor burden via pDC regulation [58].

**Fig. 2:**
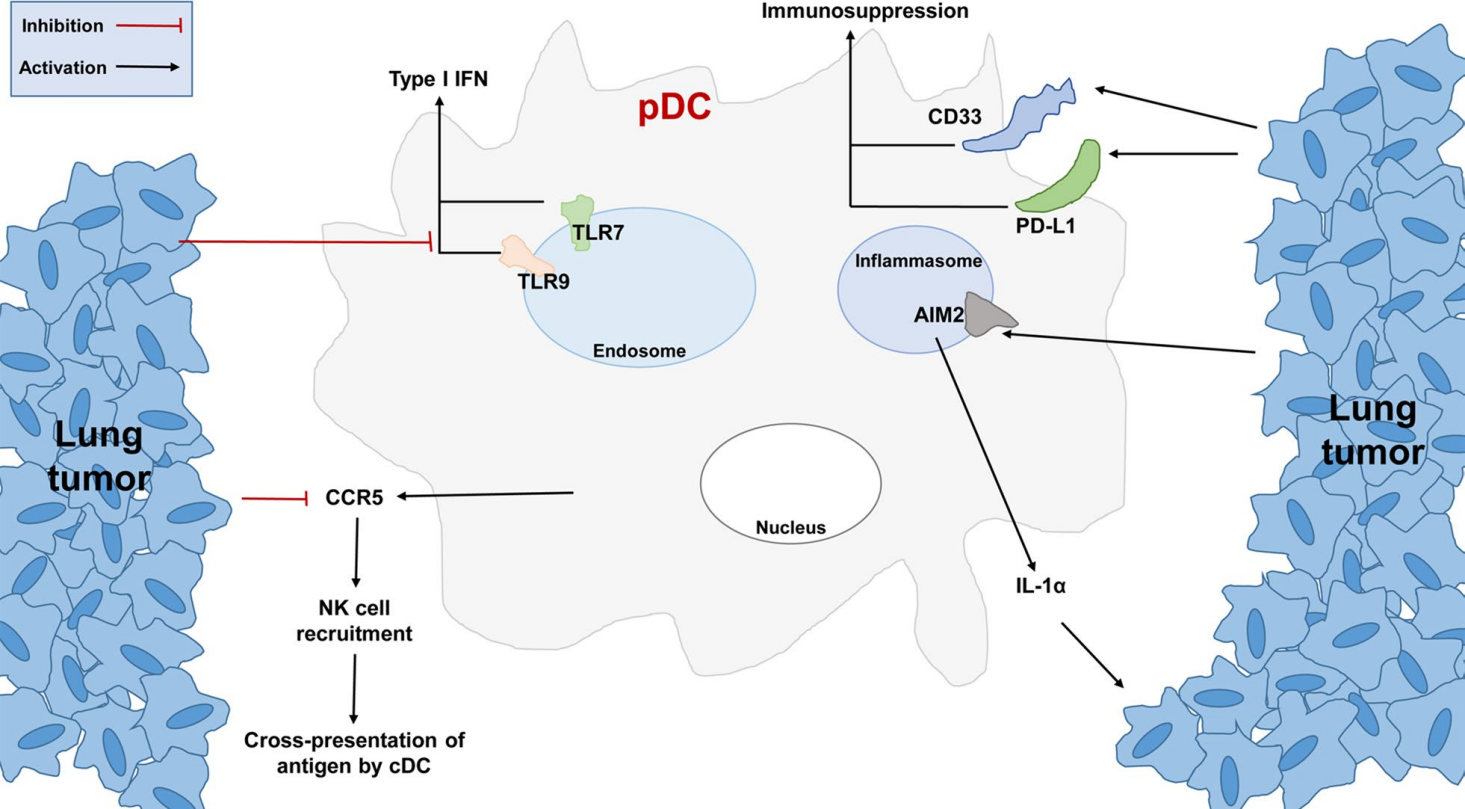
Peritumoral pDCs contribute to lung tumor progression. Impaired type I IFN secretion may faciliate tumor escape. CD33 and PD-L1 are upregulated in pDCs and contribute to immunosuppression. Lung tumors can activate AIM2 and inhibit CCR5 to construct a tumor-supportive microenvironment. pDC: plasmacytoid DC; cDC: conventional DC; NK: natural killer; IFN: interferon; CCR5: C–C chemokine receptor type 5; TLR: toll-like receptor; AIM2: melanoma 2; PD-L1: programmed cell death 1 ligand 1; IL-1α: interleukin 1α

Shi et al. [59] used flow cytometry to examine the pDC present levels in peripheral blood from 52 NSCLC patients and 52 healthy controls. Patients with higher tumor stages had higher pDC levels than those with lower stages. Rosalinda Sorrentino and colleagues [60] isolated pDCs from NSCLC tissues and found that higher percentages of immunosuppressive-phenotype pDCs were recruited in lung tumor regions with high expression of CD33 and PD-L1, which makes a contribution to the immunosuppressive tumor microenvironment. Moreover, these pDCs were able to produce high levels of interleukin 1α (IL-1α) in a melanoma 2 (AIM2)-dependent manner, which facilitated tumor cell proliferation. In many cases, the depletion of pDCs may reverse the immunosuppressive environment and decrease the lung tumor burden, thereby indicating the role of pDCs in promoting lung tumor processes. The interaction between lung tumors and pDCs is depicted in Fig. 2.

### Regulatory DCs (regDCs) are induced by lung tumors

cDCs play a fundamental role by presenting antigens during the induction of immune responses. However, the tumor cells can reprogram cDCs into Gr-1^−^ or Gr-1^+^ regDCs in tumorous lung tissue [61]. regDCs may inhibit antitumor responses and support tumor growth, thus facilitating lung carcinoma development [61]. The immunosuppressive mechanisms of regDCs have been examined in various studies. Spallanzani et al. [62] showed that regDCs silence IFN-γ secretion by NK cells via reducing IL-18 secretion and by active cell-to-cell mechanisms mediated by IL-10. These mechanisms may promote the evasion of antitumor responses. In addition, Zhang et al. [63] demonstrated that regDCs can induce a higher percentage of Tregs to maintain immune tolerance during hepatocytes transplantation. However, under tumor conditions, the outcomes induced by regDCs could impede tumor clearance.

### Downregulation of DC effector molecules in lung tumors

The unbalanced differentiation of DC precursors in TME has been demonstrated in many studies [64, 65]. After culturing DCs in the medium with serum from lung tumor patients, the expression of co-stimulatory molecules, such as CD40, CD80, CD86, MHC type II and IL-12, were disrupted. Moreover, these DCs were unable to present antigens to activate T cell responses [66]. Clinical research involving lung cancers indicated that the maturation rate of DCs in tumor tertiary lymphoid structures was significantly associated with long-term survival of patients [67].

Caronni et al. [68] demonstrated that DCs conditioned by lung TME exhibit down-regulation of soluble N-ethylmaleimide-sensitive factor accessory protein receptor, vesicle associated membrane protein3 (SNARE VAMP3), which is required for antigen cross-presentation and DC-mediate tumor rejection. Additionally, it was shown that lactic acid in TME inhibited TLR3 and stimulator of IFN genes (STING) signaling, thus inhibiting the secretion of IL-12 and IFN-I by DCs. Loss of antigen cross-presentation and cytokine secretion abilities may serve to limit the anti-tumor responses induced by DCs.

Both the nuclear factor kappa-B (NF-κB) and signal transducer and activator of transcription 3 (STAT3) pathways were suppressed in an in vitro model of DC dysfunction using NSCLC patient’s serum. Altered expression of functional cluster genes associated with DC differentiation was observed in this model [69]. This finding indicated that lung cancer cells can release soluble factors which could impair DC differentiation into the peripheral blood. As NF-κB suppression has a negative impact on DC activity, short hairpin RNA-based adjuvant targeting inhibitory proteins of NF-κB (IκBα), a suppressor of NF-κB, has been developed to activate DCs under tumor conditions [70]. Furthermore, NF-κB expression in lung tumor cells is essential for primary tumor growth. In prior studies, inhibition of the NF-κB pathway could prolong the survival rate of patients with a defined subset of lung adenocarcinomas [71]. Recent studies have also shown that blockading NF-κB signaling in the lung epithelium can prevent lung cancer development, but the efficiency of this treatment was neutralized by plasma IL-1β levels [72]. The complex mechanisms involved in NF-κB regulation have not yet been fully identified, and how this “double-edged sword” can be fully utilized in lung cancer therapies requires further investigation.

### Immunosuppressive molecules secreted by lung tumor-derived DCs

Higher immunosuppression marker expression on DCs was also detected in lung cancers compared to the healthy controls. B7-H3 is a member of the programmed death ligand (PD-L) family, which is broadly expressed on lymphoid organs with the dual functions of co-inhibiting and co-stimulating T cells. Relative to normal lung tissues, B7-H3 is up-regulated in tumor-derived DCs [23]. A blockade of B7-H3 can restore the T cell stimulatory ability of NSCLC-derived DCs, thus indicating the crucial role it plays in mediating T cell suppression by DCs under tumor conditions. Radiofrequency ablation has been shown to result in T cell proliferation and a reduction of the B7-H3^+^ DC count in the peripheral blood, further highlighting the immunosuppressive effect of this molecule under lung cancer conditions [73]. In contrast, the overexpression of B7-H3 in lung cancer tissues mediates abnormal lipid metabolism to support the development of the tumors [74]. Some researchers are trying to develop B7-H3 as a diagnostic biomarker and apply the blockade of B7-H3 as a novel therapeutic approach for the treatment of lung cancer [75, 76].

MicroRNAs (miRNAs) belong to a family of small non-coding RNAs that are individually capable of regulating cell biological pathways, many of which are involved in immune regulation and tumor development [77]. Full transcriptome sequencing of lung tumor-derived DCs revealed a set of consistently dysregulated miRNAs, such as miR-301a and miR-31 [47]. Overexpression of miRNA-301a can suppress the IL-12 secretion in DCs, while decreased expression of miRNA-301a can result in decrease IFN-γ release from antigen-specific cytotoxic T cells and delayed lung tumor growth [78]. Expression of miRNA-31 in lung cancers is related to lower survival rate, drug resistance, lymph node metastasis, and perturbation of the cell cycle [78]. miRNA-31-3p-overexpressing DCs induce pro-invasive lung cancer shape changes indicative of increased invasive behavior [78]. These results indicate that lung tumors can escape from immune surveillance, in part, by reprogramming miRNA expression in DCs.

T-cell immunoglobulin and mucin-domain 3 (Tim3) is preferentially expressed in Th1 and Th17 cells with a negative impact on immune activation [79]. However, Tim3 is also expressed in myeloid cells, including macrophages and DCs. Previous studies have shown that DCs in the lung tumor tissues have a higher expression of Tim3 than normal DCs [80]. Activation of the Tim3 pathway in DCs can suppress nucleic acids transportation, and thereby limit the nucleic acid-mediated immune response [81].

## Drivers of DC anergy in lung cancer

It is clear that lung tumors can induce various DC functional changes to promote tumor processes, but how this occurs and which specific signal or signals from the tumor can interact with DCs is still unknown. To gain information related to these phenomena, researchers have performed diverse studies on lung tumor-derived DCs. During cancer development, protein-glycan interactions can influence many processes. Galectin 1 was the first discovered protein from the glycan-binding family. Its overexpression in several tumors is related to the metastasis [82]. Recent studies have determined that higher secreted amounts of Galectin 1 by lung cancer cells can alter the phenotypes of MoDCs and impair all reactive T cell response by regulating the activity of the inhibitor of DNA binding 3 (ID3), which can induce IL-10 autocrine [83].

High mobility group box-1 protein (HMGB1) is a nuclear protein that is highly correlated with metastasis in multiple tumors) [84]. Extracellular HMGB1 can influence the recruitment and differentiation of antigen-presenting cells, thereby leading to the suppression of T cell-dependent immune response. Additionally, the upregulation of HMGB1 in NSCLC can reduce the sensitivity of tumor cells to chemotherapy regents [85–88]. Furthermore, HMGB1-mediated production of thymic stromal lymphopoietin (TSLP) by tumor cells can modulate DCs via the TSLP receptor under physiological conditions. Interaction of HMGB1 with its receptors (i.e. RAGE: receptor for advanced glycation end products, TLR2) together with the TSLP/TSLPR axis on DCs can induce the activation of Tregs and HMGB1, which can then interact with Tim3 to facilitate immunosuppression [80, 89]. In previous studies, HMGB1 inhibitors have been used for anticancer therapy and were able to reduce the percentage of Treg-activating DCs to enhance immune responses in a breast cancer model [90, 91]. How this treatment affects DC function in lung tumors, however, still requires further research. Moreover, exosomes from Lewis lung carcinomas block DC differentiation and induce cell apoptosis. They also induce the expression of PD-L1 in cDCs and play a role in DC-associated immune suppression [92].

## Progress in the clinical application of DC function restoration

**Fig. 3:**
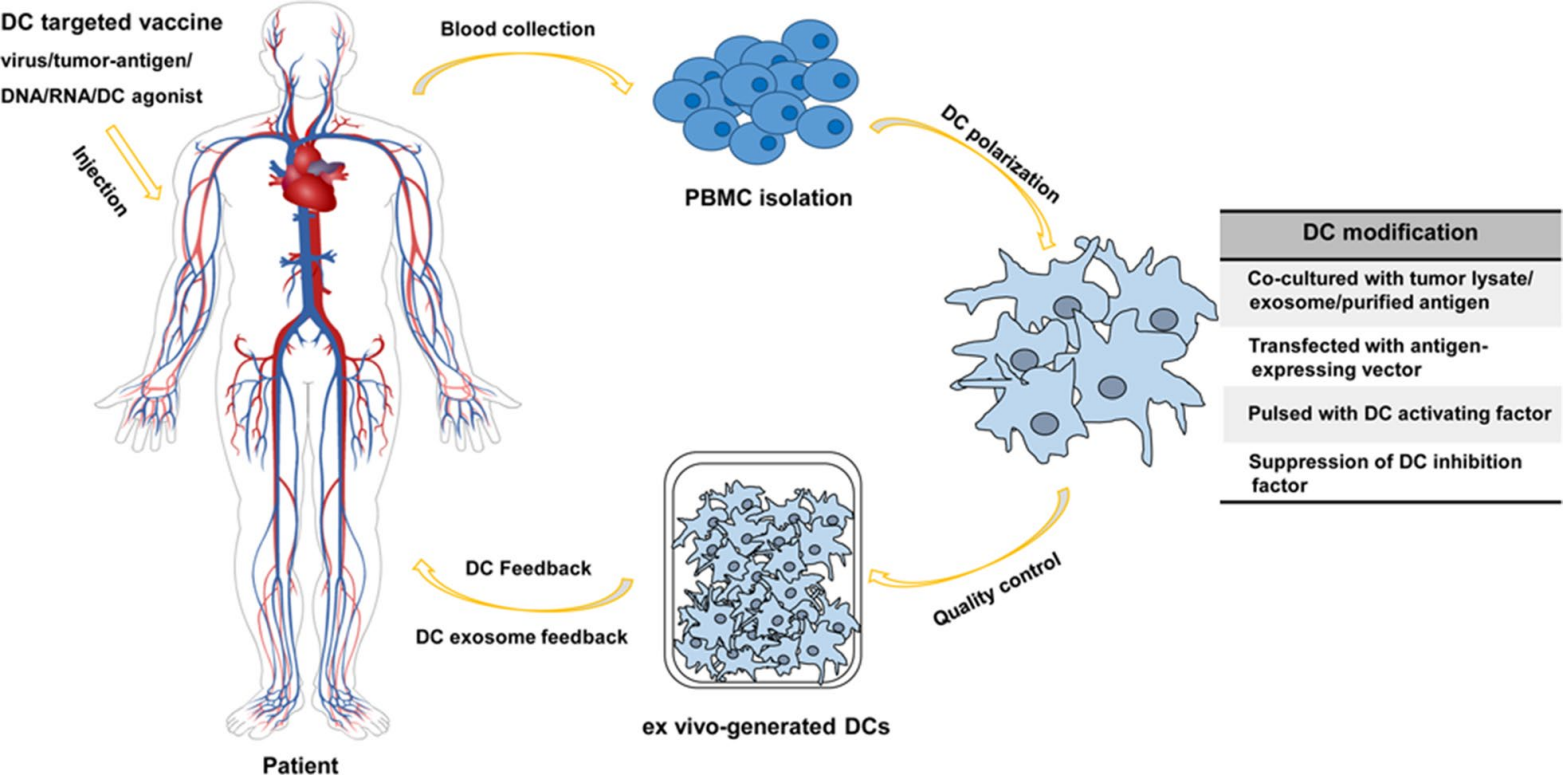
Brief procedures for DC-based immunotherapy. For DC activation in vivo, various DC agonists were injected directly into patients. For DC vaccine construction in vitro, PBMCs were isolated from patients and polarized into DC in the presence of cytokines. After modified with antigen or DC activation factors, ex vivo-generated DCs were feedback to patients to get therapeutic effects. DC: dendritic cells; PBMC: peripheral blood mononuclear cells

The first clinical trial on therapeutic cancer vaccine was carried out in 1998 on patients with melanoma [93]. Since the activation of the antigen-presenting ability of DCs is directly related with the induction of the CD8^+^ T cell response, researchers have focused on the modification of DCs in order to enhance the anti-tumor immune responses in vivo [94–96]. Many studies have demonstrated that most malignant tumors, including lung cancers, can produce a variety of factors that suppress anti-tumor immunity. Moreover, they have also shown that the maturation rate of DCs in patients with lung cancer is independently associated with survival [97, 98]. The ratio of Tregs and cancer recurrence decreases after DC vaccination combined with cytokine-induced killer (DC/CIK) immunotherapy in patients with NSCLC, indicating that this strategy is generally successful [99]. MoDCs are most widely used due to their easy acquisition from patients [100]. There are two main procedures used to activate DC-induced immunity in patients: one is to isolate DCs from patients and then feedback them after stimulation and modification, and the other is to target and activate DCs directly in vivo using specific molecules or vectors (Fig. 3).

Tumor-specific immune activation is the biggest advantage of immunotherapy compared to conventional strategies. In attempts to generate a successful DC vaccine, various antigens have been used to activate DCs, such as tumor cell lysates [101], exosomes [102], and tumor-associated antigens [103]. Significantly high expression of Survivin [104] and mucin1 (MUC1) [105] in lung cancers makes them suitable for pulsing DCs. In a phase I clinical trial involving 15 patients with NSCLC, Survivin, and MUC1 were used together to pulse DCs in vitro. Additionally, suppressor of cytokine signal 1 (SOCS1) [106] was also used in this system due to its essential role in negative regulation of DC differentiation and antigen presentation. After DC vaccine treatment, the expression of tumor markers was significantly reduced and the living quality of all patients was improved [107]. In another clinical trial, DCs were transduced with an adenoviral vector expressing C–C motif chemokine ligand 21 (CCL21), which attracts DCs and T cells by interacting with C–C motif chemokine receptor 7 (CCR7) and C-X-C motif chemokine receptor 3 (CXCR3) receptors, recruits antigen-stimulated DCs into T-cell zones in secondary lymphoid organs, and plays a key role in T-cell activation. This was performed before the intratumoral administration in patients with advanced NSCLC (*n* = 17). In response to this therapy, the treated tumors revealed enhanced CD8^+^ lymphocyte infiltration. Moreover, 6 of the 16 patients demonstrated tumor antigen-specific IFN-γ secretion and 3 patients revealed non-specific secretion of IFN-γ. Humoral responses to tumor-associated antigens were also detected in 4 of 8 patients. Moreover, after the injection of the CCL21-DC vaccine, the increased tumor expression of PD-1 was also detected [108]. As described previously, only 20% of patients respond to PD-1/PD-L1 inhibitors, whereas patients without PD-1/PD-L1 expression hardly benefit from the treatment of PD-1/PD-L1 inhibitors. This study suggests that using a combined checkpoint blockade with a DC vaccine may result in better clinical outcomes for patients with NSCLC.

Direct activation of DCs in vivo stands as another strategy for tumor therapy. As early as 1998, FLT3 ligands were used to induce MHC type II, CD11c^+^ and CD205^+^ DCs in mouse lymphoid and nonlymphoid tissues to generate effective anti-tumor responses [109]. Further studies proved that the FLT3 ligands administration after radiation therapy prolongs the survival rate of metastatic lung cancer in a mouse model through the enhancement of tumor antigen presentation by DCs [110]. In a preclinical melanoma model, a short hairpin RNA-based adjuvant that was able to silence IκBα expression was also shown to significantly activate the NF-κB pathway, thus promoting DC migration to lymph nodes and activating CD8^+^ T cells [70].

Although progress has been made in the development of DC-based immunotherapies, their therapeutic effect is still limited due to the complications presented by lung cancers. As such, employing a combination of different immunotherapy methods is currently essential in the fight against lung cancer. The immune regulatory function of lung cancers has been demonstrated in many previous studies, and further understanding on the interaction between lung cancers and DCs will be critical to prevent tumor growth in the future.

## Future prospects

**Fig. 4:**
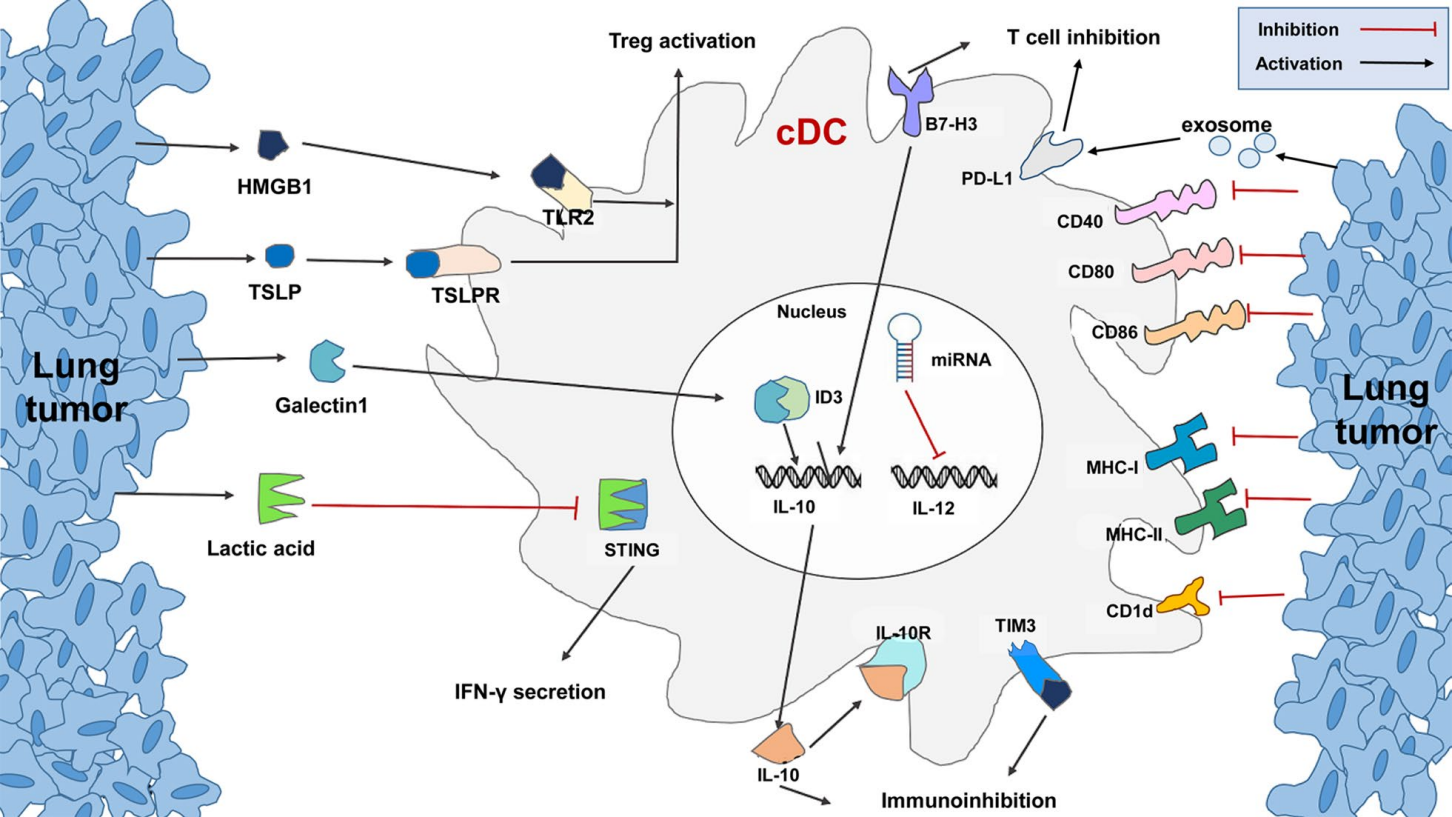
Lung tumors induce cDC anergy through different pathways. Tumors can alter the molecular expression of DC and also upregulate the immunosuppression miRNAs in DCs to inhibit the secretion of IL-12. Exosomes secreted by lung tumor cells can induce PD-L1 expression in DCs. Furthermore, different factors (HMGB1, TSLP, lactic acid) are highly expressed in lung tumors, and these may interact with different pathways within DCs to achieve a tumor-supportive microenvironment. cDC: conventional DC; HMGB1: high mobility group box-1 protein; TSLP: thymic stromal lymphopoietin; TSLPR: thymic stromal lymphopoietin receptor; IFN-γ: interferon γ; STING: stimulator of IFN genes; TLR2: toll-like receptor 2; Treg: regulatory T cells; PD-L1: programmed cell death 1 ligand 1; IL-10R: interleukin 10 receptor; TIM3: T-cell immunoglobulin and mucin-domain 3

The immunosuppressive ability of lung cancer limits the usefulness of immune control [111]. Numerous investigations have identified the different mechanisms responsible for DC anergy in lung cancers, including the downregulation of functional markers, the induction of immunosuppressive molecular expression, and the exclusion of functional cDCs from lung-tumor lesions (Fig. 4). However, current immunotherapy strategies mainly focus on enhancing the antigen-presenting activity of the DCs rather than blocking the immunosuppression induced by lung tumors [112].

Recently, one lung cancer patient was involved in the first-in-human study evaluating a DC targeting lentiviral vector which deliver antigen-encoding gene could activate antigen-specific T cell responses effectively in vivo [113]. This reminds us that this vector could also be used to deliver DC-activation genes in the future. It will be more convenient to enhance the overall DC activity directly in vivo to overcome the immunosuppression induced by lung cancer.

In spite of the therapeutic excitement of DC vaccines against lung cancer, significant challenges are still remain on their wide adoption in clinical use. Chiappori et al. [114] carried out a randomized-controlled phase II trial of salvage chemotherapy after immunization with a tumor protein p53-transfected DC vaccine in recurrent small cell lung cancer patients and found no survival differences between groups. As previously described, the injection of a tumor-antigen-loaded DC vaccine failed to consistently migrate into the lymphocyte node and elicit T-cell responses, and this may contribute to the immunosuppressive environment observed in patients [115–117]. However, there have been few clinical trials on blocking the DC-inhibition pathways in lung cancer till now. The block of common immune checkpoint PD-1/PD-L1 on DC has been partly explored. A study carried out by Chen et al. [118] demonstrated that PD-1 blockade-activated DC–CIK cells exhibit superior anti-tumor potency in several advanced solid tumors including NSCLC. Moreover, Ge et al. [119] showed that a blockade of the PD-1/PD-L1 immune checkpoint during DC vaccination induced potent protective immunity against breast cancer in hu-SCID mice. Thus, the combination of DC activation and blockage of its inhibition may generate a superior protection response in patients. It will be helpful to understand how lung cancer cells alter DC physiology and how we can generate novel immunotherapy strategies based on the powerful properties of DC. Efforts to reverse the critical elements of the immunosuppressive milieu to enhance DC vaccine potency are urgently needed in the future.

## Conclusion

Lung cancer has the highest incidence among all cancers worldwide, and DC-based immunotherapy has become an important strategy to fight this disease, mainly via the direct activation of cytotoxic T cell responses. The recent development of DC vaccine has furthered our understanding of the vital roles of DCs in the control of tumor progression. However, the complex regulation between lung tumor and DC requires rational manipulation of DCs to initiate protective immunity. To this end, a sufficient understanding of the interaction between the lung tumor and DCs will accelerate the development of new immunotherapy strategies against this fatal disease.

## Data Availability

Not applicable.

## Abbreviations

NSCLC: non-small cell lung cancer
PD-1: programmed cell death 1
PD-L1: programmed cell death 1 ligand 1
CTLA4: cytotoxic T-lymphocyte-associated antigen-4
DC: dendritic cell
TME: tumor microenvironment
TIM: tumor-infiltrating myeloid cell
cDC: conventional dendritic cell
pDC: plasmacytoid dendritic cell
MDP: monocyte-dendritic cell progenitor
CMP: common myeloid progenitor
FLT3: FMS-like tyrosine kinase 3
cMoP: common monocyte progenitor
MoDC: monocyte-derived dendritic cell
TLR: toll-like receptor
IFN: interferon
NK: natural killer
CCR: C–C chemokine receptor
Treg: regulatory T cell
LPS: lipopolysaccharides
IL: interleukin
AIM2: melanoma 2
regDC: regulatory dendritic cell
miRNA: microRNA
ID3: inhibitor of DNA binding 3
DAMP: damage-associated molecular pattern
TSLP: thymic stromal lymphopoietin
SOCS1: suppressor of cytokine signal 1
